# Human Placenta Extract (HPH) Suppresses Inflammatory Responses in TNF-α/IFN-γ-Stimulated HaCaT Cells and a DNCB Atopic Dermatitis (AD)-Like Mouse Model

**DOI:** 10.4014/jmb.2406.06045

**Published:** 2024-09-11

**Authors:** Jung Ok Lee, Youna Jang, A Yeon Park, Jung Min Lee, Kyeongsoo Jeong, So-Hyun Jeon, Hui Jin, Minju Im, Jae-Won Kim, Beom Joon Kim

**Affiliations:** 1 Department of Dermatology, College of Medicine, Chung-Ang University, Seoul 06974, Republic of Korea; 2 Department of Medicine, Graduate School, Chung-Ang University, Seoul 06973, Republic of Korea; 3 Research and Development Center, Green Cross Wellbeing Corporation, Gyeonggi-do 16950, Republic of Korea

**Keywords:** Human placenta Hydrolysate (HPH), Atopic dermatitis (AD), immunized splenocytes, filaggrin (FLG), T helper 2 (Th2)

## Abstract

Atopic dermatitis (AD), a chronic inflammatory disease, severely interferes with patient life. Human placenta extract (HPH; also known as human placenta hydrolysate) is a rich source of various bioactive substances and has widely been used to dampen inflammation, improve fatigue, exert anti-aging effects, and promote wound healing. However, information regarding HPH’s incorporation in AD therapies is limited. Therefore, this study aimed to evaluate HPH’s effective potential in treating AD using tumor necrosis factor (TNF)-α/interferon (IFN)-γ-stimulated human keratinocytes (HaCaT), immunized splenocytes, and a 2,4-dinitrochlorobenzene (DNCB)-induced AD mouse model. In TNF-α /IFN-γ-stimulated HaCaT cells, HPH markedly reduced the production of reactive oxygen species (ROS) and restored the expression of nuclear factor erythroid 2-related factor 2 (Nrf2), superoxide dismutase 1(SOD1), catalase, and filaggrin (FLG). HPH reduced interleukin (IL)-6; thymus- and activation-regulated chemokine (TARC); thymic stromal lymphopoietin (TSLP); and regulated upon activation, normal T cell expressed and presumably secreted (RANTES) levels and inhibited nuclear factor kappa B phosphorylation. Additionally, HPH suppressed the T helper 2 (Th2) immune response in immunized splenocytes. In the AD-like mouse model, it significantly mitigated the DNCB-induced elevation in infiltrating mast cells and macrophages, epidermal thickness, and AD symptoms. HPH also reduced TSLP levels and prevented FLG downregulation. Furthermore, it decreased the expression levels of IL-4, IL-5, IL-13, TARC, RANTES, and immunoglobulin E (IgE) in serum and AD-like skin lesion. Overall, our findings demonstrate that HPH effectively inhibits AD development and is a potentially useful therapeutic agent for AD-like skin disease.

## Introduction

Atopic dermatitis (AD) is a chronic inflammatory skin disorder with several symptoms, such as severe itching, erythema, dryness, and relapsable eczematous lesions [1, 2]. It is caused by a combination of complex interactions among genetic and environmental factors, the skin barrier, and immune dysregulation [3, 4]. From a pathophysiological perspective, AD is reportedly caused by an immune imbalance in the T helper 2 (Th2)-dominant environment. Consequently, B cells that accelerate serum immunoglobulin E (IgE) secretion are stimulated by the overproduction of Th2 cytokines, thus accelerating serum IgE secretion, which activates immune cells, such as neutrophils and mast cells, to induce skin tissue infiltration and the inflammatory response, further damaging the skin barrier [5-7]. Eventually, the damaged skin barrier renders it easy for various stimuli and antigens to infiltrate the skin, intensifying immune responses [8].

The skin is largely divided into three layers: the epidermis, dermis, and subcutaneous layer [9]. The epidermis, the outermost layer, is the body’s first barrier, protecting it from external hazards, such as ultraviolet radiation, injury, and pathogens [10]. It comprises keratinocytes, which constitute 90–95% of epithelial cells; melanocytes; and rare Merkel cells [11]. Keratinocytes produce chemokines, including regulated on activation, normal T cell expressed and secreted (RANTES/CCL18); thymus- and activation-regulated chemokine (TARC/CCL17); and inflammatory cytokines, such as thymic stromal lymphopoietin (TSLP), interleukin (IL)-4, IL-25, and IL-33, which contribute to inflammatory responses in the skin [12].

The dysregulation of keratinocyte function can lead to various inflammatory skin disorders, such as AD and psoriasis, by triggering an inflammatory cascade of immune cells in the skin [13]. Therefore, an immune imbalance in keratinocytes has been implicated in AD pathogenesis [14]. The skin barrier is not merely a physical barrier against the external environment but also an active part of the immune response. Therefore, to treat and prevent AD, maintaining a healthy skin barrier is crucial.

Completely curing AD is challenging. Although the patient’s condition may exhibit improvement, such improvement is frequently transient, and AD may recur. To date, topical glucocorticoids (*e.g.*, dexamethasone [DEX]), calcineurin inhibitors (*e.g.*, cyclosporine), antihistamines, and Janus kinase inhibitors (*e.g.*, upadacitinib and abrocitinib) are utilized as therapeutics for AD [15-17]. Although these medications may relieve certain symptoms, they potentially cause diverse side effects, such as bleeding, hypertension, and liver and kidney toxicity [18, 19].

DEX is extensively used owing to its potent anti-inflammatory properties [20]. Despite its effectiveness, its long-term use has been associated with significant side effects, including weight loss and muscle dysfunction and atrophy [21, 22]. Therefore, safe drugs that have few side effects and can be put to long-term use are urgently required to treat patients with AD and to enhance their quality of life.

Human Placenta Hydrolysate (HPH) is a rich source of various bioactive substances, including peptides, amino acids, enzymes, trace elements, minerals [23]. It has been shown to possess various therapeutic properties, such as regenerative, antioxidant, and anti-inflammatory effects, including promoting the effect of hair growth in telogenic mice [24], liver regeneration [25], ligament healing [26], anti-apoptotic effects on hepatocyte toxicity [27], and antiviral effects against severe acute respiratory syndrome coronavirus 2 [28]. In addition, Kwan-hoo Lee *et al*. demonstrated that HPH exhibits significant anti-inflammatory effects in both in vitro and in vivo models. Specifically, they found HPH to alleviate inflammation in lipopolysaccharide (LPS)-stimulated RAW264.7 macrophages. Additionally, they revealed that HPH effectively mitigates inflammation in rat models induced by acetic acid and carrageenan [29], suggesting that it may be potentially effective in treating AD.

Therefore, we investigated the effect of HPH on AD using TNF-α/IFN-γ-stimulated human epidermal keratinocyte (HaCaT) and 2,4-DNCB-induced AD mouse models.

## Materials and Methods

### Preparation of Human Placenta Hydrolysate (HPH)

HPH (Laennec) was manufactured by GC Wellbeing Co., Ltd., (Republic of Korea). Briefly, it was prepared via placental hydrolysis with HCl and pepsin. The final product was in liquid form and stored in 2-ml ampules.

### Cell culture and Viability Assay

HaCaT cells were purchased from the Korea Cell Line Bank (Republic of Korea). The cells were cultured in Dulbecco’s modified Eagle medium containing 10% fetal bovine serum (FBS; Gibco, USA) and 1% penicillin–streptomycin (Gibco) at 37°C in a 5% CO_2_ incubator. The viability of HaCaT cells treated with HPH and DEX (Sigma, USA) was measured using the Cell Counting Kit-8 (CCK-8) assay (Dojindo Laboratories, Japan). Briefly, HaCaT cells were seeded in 96-well plates at a density of 5 × 10^4^ cells/well and incubated at 37°C for 24 h in a 5%CO_2_ incubator. Thereafter, 40% HPH was processed down to 2.5% in two-fold dilutions, and DEX was used at 1 μM for 24 h. After cell treatment, the CCK-8 reagent was added, and the plates were incubated at 37°C for 30 min in a 5% CO_2_ incubator. The absorbance of the solution in each well was measured at 450 nm using a microplate reader (i3x; Molecular Devices).

### Measurement of Intracellular Reactive Oxygen species (ROS) Levels

Intracellular ROS production was measured using the Cellular ROS Detection Assay Kit (cat. no. ab113851, Abcam). Cells were pretreated with various concentrations of HPH for 6 h, washed with 1× assay buffer (Abcam), and incubated in 20 μM 2,7-dichlorofluorescin diacetate (DCFDA) solution (Abcam) (0.1 ml) for 45 min at 37°C with 5% CO_2_ in the dark. Subsequently, the cells were exposed to TNF-α + IFN-γ (10 ng/ml, each) and incubated for 2 h in complete medium containing 10% FBS but lacking phenol red (WelGENE, Inc.). The samples were observed using a fluorescence microscope (DMi8; Leica Microsystems GmbH), and fluorescence readings were obtained using a spectrophotometer (SpectraMax 340; Molecular Devices, Inc.) at wavelengths of 485 and 535 nm.

### Western Blot Analysis

Skin and cell proteins were extracted using RIPA lysis buffer (Thermo Fisher Scientific, USA), and the protein content was quantified using Bradford reagent (Sigma). Equal amounts of protein were separated on an 8%sodium dodecyl sulfate–polyacrylamide gel electrophoresis gel and transferred to nitrocellulose membranes (Cytiva, UK). Subsequently, the membranes were blocked with 5% skim milk in Tris-buffered saline containing 0.1% Tween-20 and probed overnight at 4°C with primary antibodies against Nrf2 Thermo Fisher Scientific, 1:5,000), superoxide dismutase type 1 (SOD1; Santa Cruz Biotechnology, USA, 1:5,000), catalase (Santa Cruz Biotechnology, 1:5,000), nuclear factor kappa B (NF-κB; Cell Signaling Technology, Inc., 1:5,000), TSLP (Abcam, 1:5,000), filaggrin (FLG; Thermo Fisher Scientific, 1:5,000), cyclooxygenase-2 (Cell Signaling Technology, 1:5,000), and inducible nitric oxide synthase (iNOS; Cell Signaling Technology, 1:5,000). Afterwards, the membranes were incubated with horseradish peroxidase (HRP)-conjugated anti-mouse (Vector Labs, USA) or anti-rabbit (Vector Labs) secondary antibodies at room temperature (RT) for 1 h. Immunodetection was performed using an Amersham ECL Kit (GE Healthcare, USA) according to the manufacturer’s protocol. The protein bands were visualized using a ChemiDoc MP Imaging System (Bio-Rad Laboratories) and analyzed using Image J software (NIH Image, USA).

### Induction of Th2-Dominant Immune Responses and Cell Culture

Female, 6-week-old BALB/c mice were purchased from Raonbio (Republic of Korea) and acclimated for 6 days. Immunized splenocytes were obtained with reference to a relevant article [30]. In brief, 1.5 ml of liquid aluminum hydroxide (13 mg/ml; Sigma), 10 mg of ovalbumin (OVA; Sigma), and 0.46 ml of phosphate-buffered saline (PBS; Gibco) were reacted at RT for 20 min; the resulting mixture (0.2 ml/mouse) was intraperitoneally injected. On day 6, the mice were boosted with the same volume of the mixture. On day 13, mouse spleens were harvested and crushed, and immunized splenocytes were collected in Roswell Park Memorial Institute (RPMI)-1640 medium. Splenocytes isolated from the mice were seeded at 4 × 10^6^ cells/well in 24-well plates. Moreover, the cells were incubated at 37°C for 7 days in a 5% CO_2_ incubator in the presence or absence of OVA (100 μg/ml), HPH (50 μl), or DEX (50 μl), and the cell supernatant was collected from each well.

### Experimental Mice

Six-week-old male BALB/c mice were purchased from Raonbio and housed in a controlled environment (temperature, 22 ± 3°C; relative humidity, 55 ± 5%; 12/12 h light/dark cycle) with free access to food (Teklad Global 18% Protein Rodent Diet; Envigo, USA) and tap water. All experimental procedures were performed according to the protocol approved by the Green Cross Wellbeing Animal Ethics Committee (approval number: A-GCWB210_20220627). The mice were euthanized and their spleens harvested. At the end of the experiment, the mice were anesthetized with isoflurane, and their blood was immediately collected from their venous vessels. The blood was kept at RT for 1 h and subsequently centrifuged at 1,000 ×*g* and 4°C for 10 min to separate the serum.

### Animal Experimental Design

Mice were randomly divided into normal and AD groups. In the AD group, AD was induced using DNCB (Sigma-Aldrich). Their dorsal hair was removed using an animal clipper, and hair removal cream was applied to completely remove fuzzy hairs. To allow the healing of micro scars from hair removal, the animals were rested for 1 day prior to AD induction. DNCB was dissolved at 1% in a 3:1 mixture with acetone (Republic of Korea) and olive oil (Duksan). Sensitization was performed by repeatedly applying the combined mixture (200 μl) to the dorsal area of the mice twice/week for a week. Thereafter, based on skin lesion observation and serum IgE levels, the BALB/c mice were finally subdivided into five groups: the saline (vehicle, *n* = 7), exclusive DNCB treatment (sham, n =7), DNCB + 0.2 ml HPH (HPH 200, *n* = 7), DNCB + 0.4 mL HPH (HPH 400, *n* = 7), and DNCB + 10 μg/ml DEX (DEX, *n* = 7) groups. From week 2, 0.5% DNCB was applied to the dorsal area three times/week for 3 weeks to continue AD induction. In addition to 0.5% DNCB-based AD induction, HPH (200 or 400 μl; subcutaneous injection twice/week) and DEX (10 mg/kg; intraperitoneal injection three times/week) were administered. The in vivo study schedule is shown in Fig. 4A.

### Scoring of Dermatitis Severity

Dermatitis scores were estimated according to previously described methods [31]. Briefly, to assess the severity of AD symptoms, three items (erythema, dryness, and scarring) were selected, and the total score was computed. Each item was scored as follows: 0 (none), 1 (mild), 2 (moderate), and 3 (severe). The minimum total score was 0 and the maximum possible score 9.

### Enzyme-Linked Immunosorbent Assay (ELISA)

IgE levels were measured using commercial kits purchased from BD Pharmingen (USA) according to the manufacturer’s instructions. Absorbance was measured using a microplate reader (i3x; Molecular Devices). The levels of the cytokines IL-1β, IL-2, IL-4, IL-5, IL-6, IL-13, and TNF-α (Merck Millipore) measured using a multiplex ELISA kit according to the manufacturer’s instructions. Median fluorescence intensity was measured using a Luminex reader (USA).

### RNA Isolation and Real-Time Quantitative Polymerase Chain Reaction (qRT-PCR) Analysis

During in vitro experimentation, HaCaT cells were seeded in 24-well plates at a density of 1.5 × 10^5^ cells/well and incubated at 37°C and 5% CO_2_ for 24 h. The cells were co-treated with 10 ng/mL each of TNF-α (BD Pharmingen) + IFN-γ (BD Pharmingen). In the *in vivo* experiment, dorsal skin was obtained after the mice had been euthanized. Dorsal skin (35–50 mg) was homogenized using liquid nitrogen. The cells and tissues were processed using the RNeasy Mini Kit (Qiagen, USA) to extract total RNA according to the manufacturer’s protocol. Complementary DNA was synthesized using a reverse transcription kit (Applied Biosystems, USA). qRT-PCR was performed using Quant Studio 3 (Applied Biosystems) with SYBR Green master mix (Applied Biosystems). The sequences of the primers are provided in Table 1. The mRNA expression levels were normalized using *GAPDH* and *β-actin* as the housekeeping genes and calculated using the 2^−ΔΔCt^ method.

### Immunohistochemistry

Skin biopsies were fixed in 10% formalin for 24 h. Paraffin-embedded 3-μm-thick sections were cut, mounted on Polysine Slides (Thermo Fisher Scientific, Inc.), dewaxed in xylene, and subsequently dehydrated in an ethanol series. The sliced sections were subjected to antigen retrieval using Tris-EDTA at 4°C for 15 min, incubated with Bloxall blocking solution (Vector Laboratories, Inc.) at RT for 30 min, and subsequently incubated overnight at 4°C with primary antibodies, including FLG, and TSLP antibodies. Thereafter, the slides were incubated with HRP using the ImmPRESS Excel Amplified Polymer Staining Kit (Vector Laboratories, Inc.). The staining was developed using the 3,3'-diaminobenzidine peroxidase substrate kit (Vector Laboratories, Inc.). To identify nuclei, the slides were counterstained with hematoxylin at RT for < 1 min. The stained slides were photographed using a slide scanner (Pannoramic MIDI; 3DHISTECH, Ltd.), observed using Case Viewer software (V 2.7; 3DHISTECH Ltd.), and analyzed using ImageJ software (V1.8.0; National Institutes of Health).

### Histological Analysis

After the mice had been euthanized, their dorsal skin was obtained to perform histological analysis. The skin was fixed in 4% paraformaldehyde and sliced into 4-μm-thick sections. The slices were stained with hematoxylin and eosin (H&E), toluidine blue (TB), and F4/80 (Santa Cruz Biotechnology, USA), and images of the stained slices were obtained using a microscope (×200). Epidermal thickness and the number of mast cells were estimated in six areas for each mouse using i-Solution Lite (iMTechnology).

### Body, Spleen, and Muscle Weights

Mice were weighed weekly throughout the experiment. At the end of the experimental period, the mice were euthanized and their spleens harvested. Spleen weight was normalized to the body weight of each individual mouse. The calf muscles, soleus (slow-twitch fibers), and quadriceps (fast-twitch fibers) were harvested, and muscle mass was calculated by dividing the weights of the soleus and quadriceps by the body weight.

### Statistical Analysis

The results are presented as the mean ± standard deviation (SD). Statistically significant differences between groups were determined using one-way analysis of variance and Tukey’s post-hoc test (SPSS 25, USA). The data are presented as follows: # *p* < 0.05, ## *p* < 0.01, and ### *p* < 0.001 compared with the normal; * *p* < 0.05, **p < 0.01, and *** *p* < 0.001 compared with the control. The *p*-values for all results are listed in Table S1.

## Results

### HPH Suppresses Oxidative Stress in TNF-α/IFN-γ-Stimulated HaCaT Cells

**Fig. 1:**
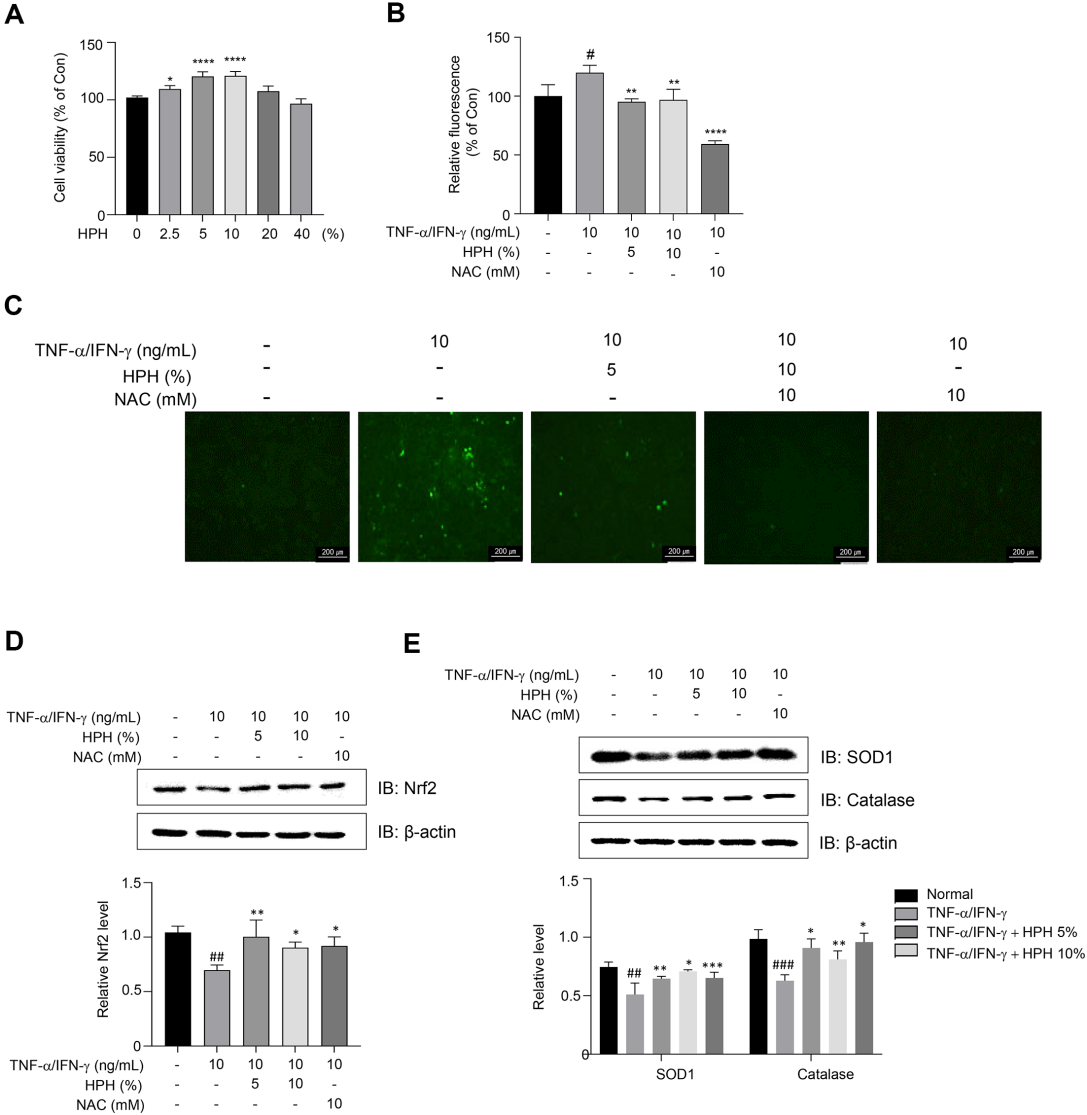
Protective effect of HPH in TNF-α/IFN-γ-stimulated HaCaT human keratinocytes. (**A**) Cytotoxicity of HPH to HaCaT cells. HPH did not exhibit any cytotoxicity at concentrations of up to 40%. (**B**) Intracellular ROS generation and analysis of ROS levels with and without TNF-α/IFN-γ stimulation via (**C**) fluorescence microscopy. Increases in Nrf2 (**D**), SOD1, and catalase (**E**) levels by HPH in TNF-α/IFN-γ-stimulated HaCaT keratinocytes according to Western blot analysis. Data are expressed as the mean ± S.D. *p < 0.05, **p < 0.01, ***p < 0.001, ****p < 0.0001 compared with the control group. ^#^*p* < 0.05, ^##^*p* < 0.01, ^###^*p* < 0.001, ^####^*p* < 0.0001 compared with the normal group.

The cytotoxicity of HPH was evaluated using the WST-8 assay on HaCaT cells exposed to varying concentrations of HPH (range: 2.5%–40%) for 24 h. The results indicated that HPH concentrations of up to 40%did not exert any cytotoxic effects on HaCaT cells. Furthermore, HPH concentrations within the 2.5%–10% range enhanced the proliferation of HaCaT cells (Fig. 1A). It is well known that ROS promotes inflammation in a variety of diseases, such as diabetes, inflammatory bowel disease (IBD), chronic obstructive pulmonary disease (COPD), and osteoarthritis (OA) [32, 33]. The DCFDA assay results, obtained using a microplate reader, indicated an increase in intracellular ROS levels in TNF-α/IFN-γ-stimulated HaCaT cells, which was substantially mitigated in a dose-dependent manner upon HPH treatment (Fig. 1B). The TNF-α/IFN-γ-stimulated HaCaT cells produced high fluorescence intensity, indicating an increase in intracellular ROS levels, whereas HPH treated cells resulted in decreased fluorescence intensity. Furthermore, flow cytometry analysis of DCFDA-stained cells corroborated these fin dings, showing increased intracellular ROS levels in TNF-α/IFN-γ-stimulated HaCaT cells, which were subsequently reduced with HPH treatment. N-acetyl cysteine served as a positive control for ROS scavenging (Fig. 1C). To ascertain whether HPH affects the Nrf2 signaling pathway, Nrf2 levels were assessed via Western blot analysis. As shown in Fig. 1D, Nrf2 was downregulated following cellular stimulation with TNF-α/IFN-γ but significantly upregulated after HPH treatment. Moreover, TNF-α/IFN-γ stimulation decreased the expression of SOD1 and catalase, while HPH treatment increased SOD1 and catalase expression compared to TNF-α/IFN-γ-stimulated HaCaT cells, indicating a cytoprotective effect (Fig. 1E).

### HPH Downregulates the Expression of Inflammatory Cytokines and Chemokines in TNF-α/IFN-γ-Stimulated HaCaT Cells Keratinocytes

**Fig. 2:**
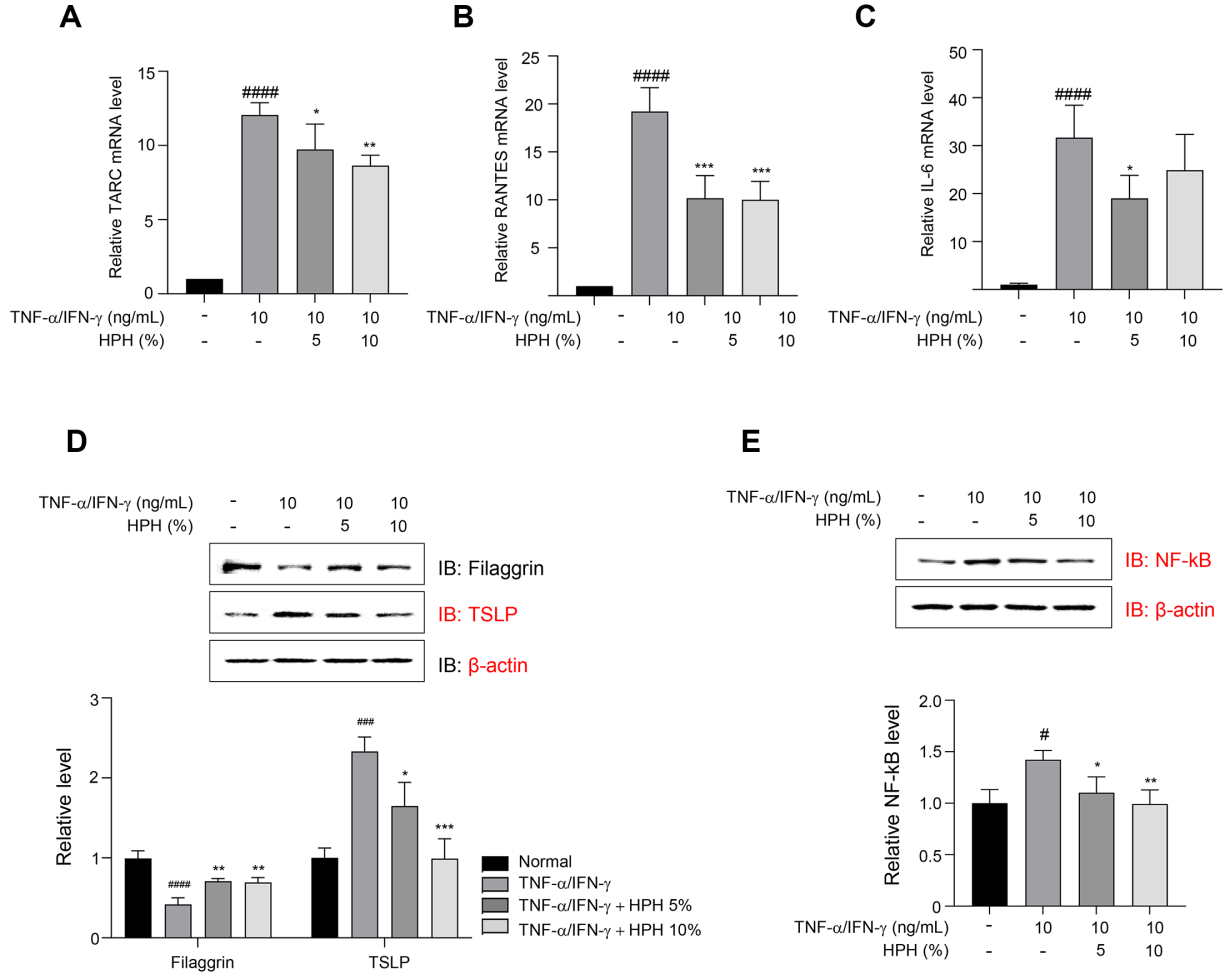
Cell viability and oxidative stress chemokines and growth factors in TNF-α/IFN-γ-stimulated human keratinocytes. (**A**) TARC, (**B**) RANTES, and (**C**) IL-6 mRNA expression levels were measured using qRT-PCR. (**D**) The expression of filaggrin and TSLP by HPH in TNF-α/IFN-γ-stimulated HaCaT keratinocytes according to Western blot analysis. (**E**) The expression of NF-kB by HPH in TNF-α/IFN-γ-stimulated HaCaT keratinocytes according to Western blot analysis. Data are expressed as the mean ± S.D. *p < 0.05, **p < 0.01, ***p < 0.001 compared with the control group. ^#^*p* < 0.05, ^###^*p* < 0.001, ^####^*p* < 0.0001 compared with the normal group.

To investigate the anti-inflammatory effects of HPH in TNF-α/IFN-γ-stimulated HaCaT cells, we analyzed the mRNA expression levels of TARC, RANTES (also known as CCL5), and IL-6. TNF-α/IFN-γ stimulation upregulated the mRNA expression levels of TARC, RANTES, and IL-6 compared to the control. However, treatment with HPH markedly downregulated these expression levels (Fig. 2A-2C). Filaggrin (FLG) reduction, a hallmark of AD skin lesions associated with skin barrier dysfunction, was effectively inhibited by HPH in TNF-α/IFN-γ- stimulated HaCaT cells (Fig. 2D). Furthermore, HPH suppressed the TNF-α/IFN-γ-mediated TSLP increase compared to the control. In addition, HPH reduced the production of TNF-α/IFN-γ-induced phosphorylated NF-κB-p65 in a concentration dependent manner (Fig. 2E).

### HPH Restores the Th1/Th2 Imbalance in Mouse Splenocytes

**Fig. 3:**
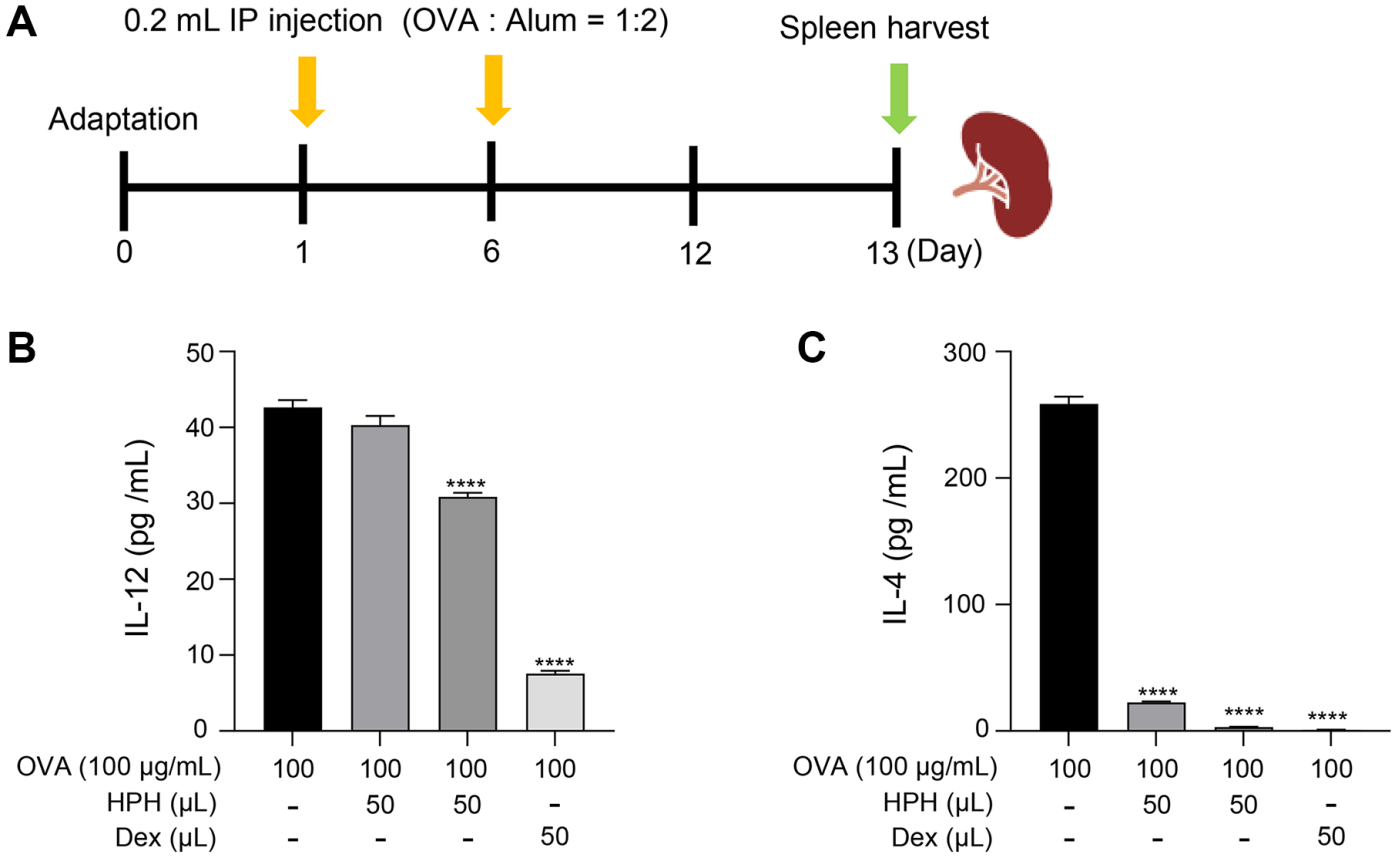
Effect of HPH on Th1/Th2 cytokine balance in OVA-induced mouse splenocytes. (**A**) To obtain immunized splenocytes, OVA was administered on days 1 and 6, and the spleen was harvested on day 13. (**B**) The IL-12 (Th1 cytokine)/IL-4 (Th2 cytokine) ratio with respect to the sample treatment in immunized splenocytes was calculated. IL-12 and IL-4 levels in cell culture supernatants were measured using ELISA. Data are expressed as the mean ± S.D. ^#^*p* < 0.05, ^####^*p* < 0.0001 compared with the normal group.

The primary pathogenesis of AD is characterized by an imbalance in the Th1/Th2 immune response, with a predominance of Th2 cell differentiation. To investigate the AD-suppressive effect of HPH on mouse splenocytes, an ex vivo experiment was conducted using mouse splenocytes predisposed to a Th2 cell response. As shown in Fig. 3A, spleens were harvested from mice immunized with OVA to isolate splenocytes. Splenocytes from immunized mice exhibited significantly elevated levels of Th1 cytokines (IL-12) and Th2 cytokines (IL-4). HPH treatment reduced IL-12 and IL-4 levels (Fig. 3B and 3C). Therefore, HPH may restore the balance between the Th1 and Th2 helper T cell types and potentially suppress AD.

### Inhibitory Effects of HPH on AD-Like Symptoms in DNCB-Sensitized Mice

**Fig. 4:**
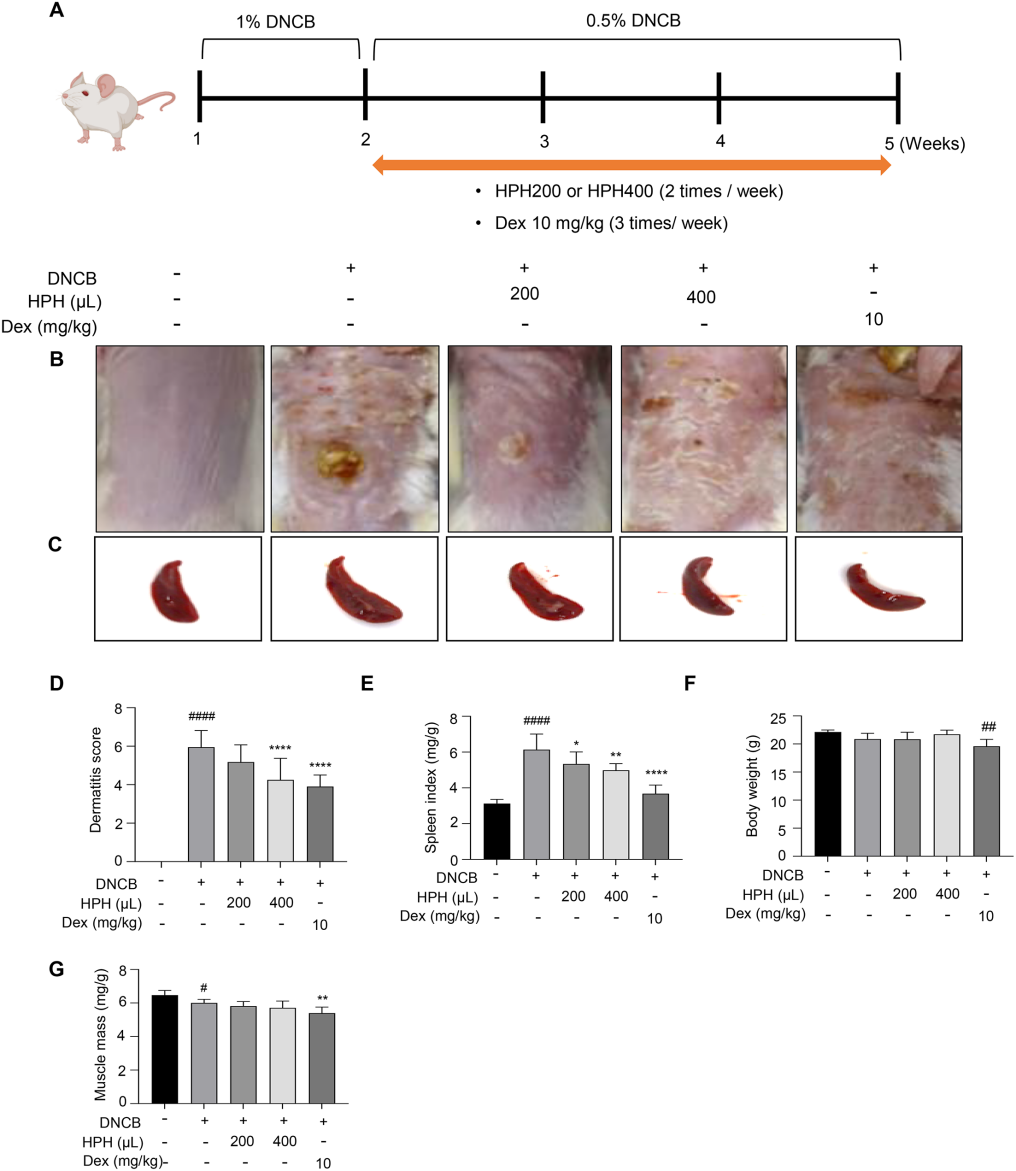
Effect of HPH on skin lesions and spleen changes in DNCB-sensitized BALB/c mice. (**A**) BALB/c mice were sensitized to DNCB (1% or 0.5%) three times/week for 4 weeks. HPH (200 or 400 μL) was subcutaneously injected twice/ week, and DEX (positive control group; 10 mg/kg) was intraperitoneally injected three times/week. (**B**) Images were taken at the end of the experiment. The images display representative dorsal skin lesions in each group. (**C**) Images of spleens harvested from BALB/c mice are shown. (**D**) The dermatitis score reflecting erythema, dryness, and scarring in skin lesions of individual mice was assessed. (**E**) For each mouse, the body weight-adjusted spleen weight was quantified. (**F**) The body weight of each group. (**G**) For each mouse, the calf muscle was harvested and the body weight-adjusted muscle mass quantified. Data are expressed as the mean ± S.D. *p < 0.05, **p < 0.01, ****p < 0.0001 compared with the control group. ^#^*p* < 0.05, ^####^*p* < 0.0001 compared with the normal group. N, normal group; C, DNCB-induced control group; HPH200, HPH 200 μL-administered group; HPH400, HPH 400 μL-administrated group; DEX, dexamethasone (10 mg/kg)-administered group.

An animal model was developed using BALB/c mice sensitized with DNCB. HPH was administered at dosages of 200 μl or 400 μl for 3 weeks, twice weekly (Fig. 4A). Physiological symptoms of AD, such as erythema, dryness, and scarring, markedly increased in the DNCB-treated group compared to the naive group. In contrast, the HPH400 and DEX treated groups showed significant improvement in AD symptoms on the dorsal skin (Fig. 4B and 4D). Both the HPH200 and HPH400 groups displayed significantly reduced spleen weights compared to the DNCB-induced AD group (Fig. 4C and 4E). However, body weight exclusively decreased in the DEX-treated AD group by the end of this experiment (Fig. 4F). Notably, muscle mass in the DEX group decreased by approximately 10% compared to the naive group, whereas no change in muscle mass was observed in the HPH200 and HPH400 groups (Fig. 4G). These findings suggest that HPH has the potential to alleviate AD symptoms while minimizing long-term side effects, such as muscle atrophy, commonly associated with existing AD treatments.

### Inhibitory Effects of HPH on Immune Cell Infiltration in DNCB-Sensitized Mice

**Fig. 5:**
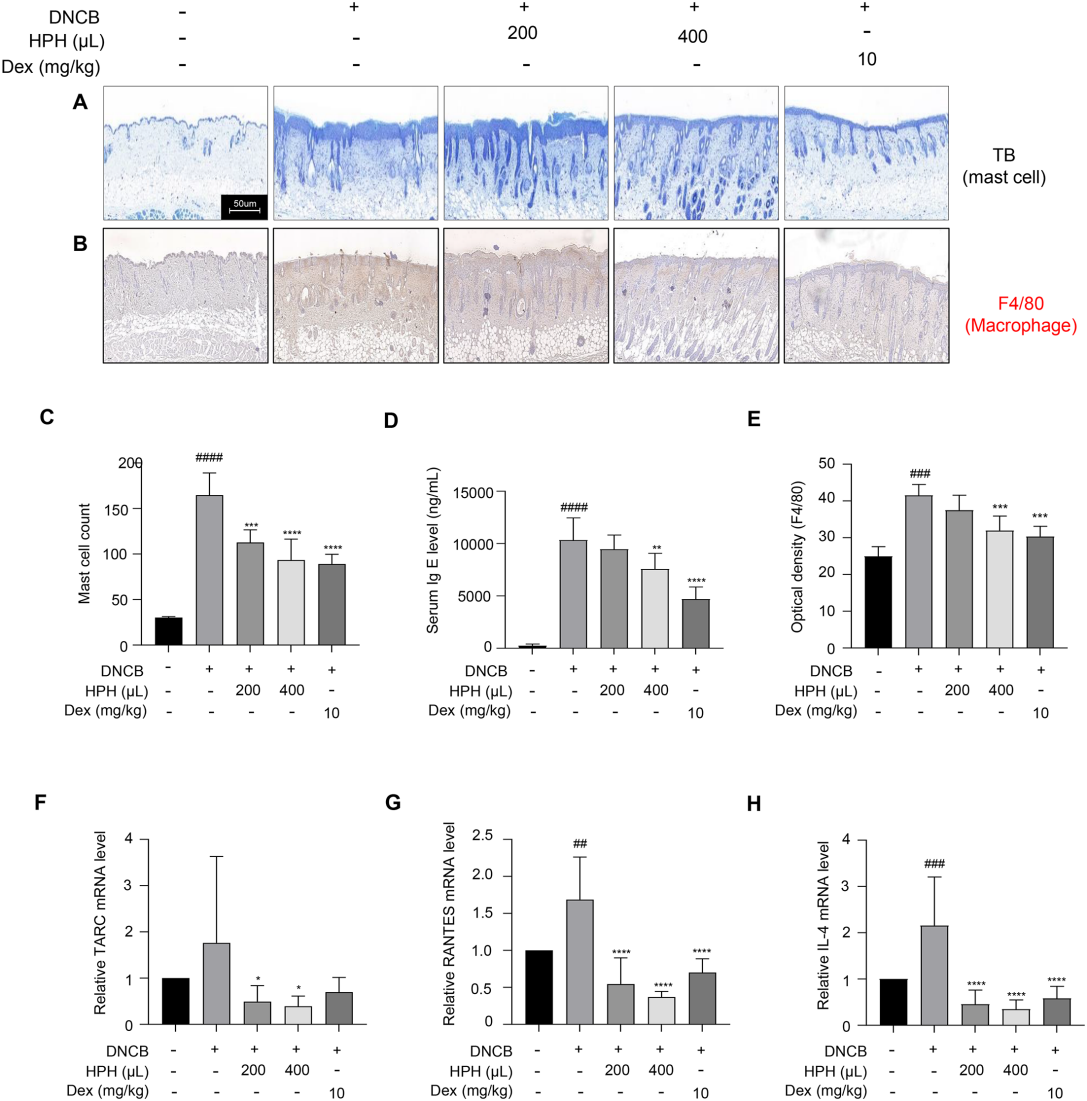
Effect of HPH on mast cell infiltration, IgE level, and macrophage level in the dorsal skin tissues of DNCB-sensitized BALB/c mice. After autopsy, dorsal skin tissue samples were obtained from individual mice from each group. (A and B) Dorsal skin tissue sections were stained with TB and anti-F4/80 antibodies. (**C**) Mast cell number. (D Serum IgE level. (**E**) Optical density of F40/80. The graphs show mean values. RNA was extracted from the dermal skin tissue of DNCB-induced BALB/c mice. (**F**) TARC, (**G**) RANTES, (**H**) and IL-4 mRNA expression levels were analyzed using qRT-PCR. Data are expressed as the mean ± S.D. *p < 0.05, **p < 0.01, ***p < 0.001, ****p < 0.0001 compared with the control group. ^##^*p* < 0.01, ^###^*p* < 0.001, ^####^*p* < 0.0001 compared with the normal group.

The numbers of mast cells in the AD skin lesions of DNCB-treated mice were assessed. TB staining revealed that DNCB increased dermal mast cell numbers, which were significantly reduced in a dose-dependent manner in the HPH treated groups (Fig. 5A and 5C). Serum IgE levels also decreased in HPH-treated mice (Fig. 5D). Macrophages play a crucial role in the immune system and host defense during the inflammatory phase via inflammatory cytokine release. The F4/80 antigen, a surface glycoprotein frequently expressed on macrophages, showed decreased expression in the HPH 200 and HPH 400-treated AD-like skin lesions compared to the exclusive DNCB treatment group (Fig. 5B and 5E). In addition, HPH inhibited TARC, RANTES, and IL-4 mRNA expression levels in AD-like skin lesions compared to exclusive DNCB treatment (Fig. 5F-5H).

### HPH-Induced Recovery of Cytokine Levels in DNCB-Sensitized Mice

**Fig. 6:**
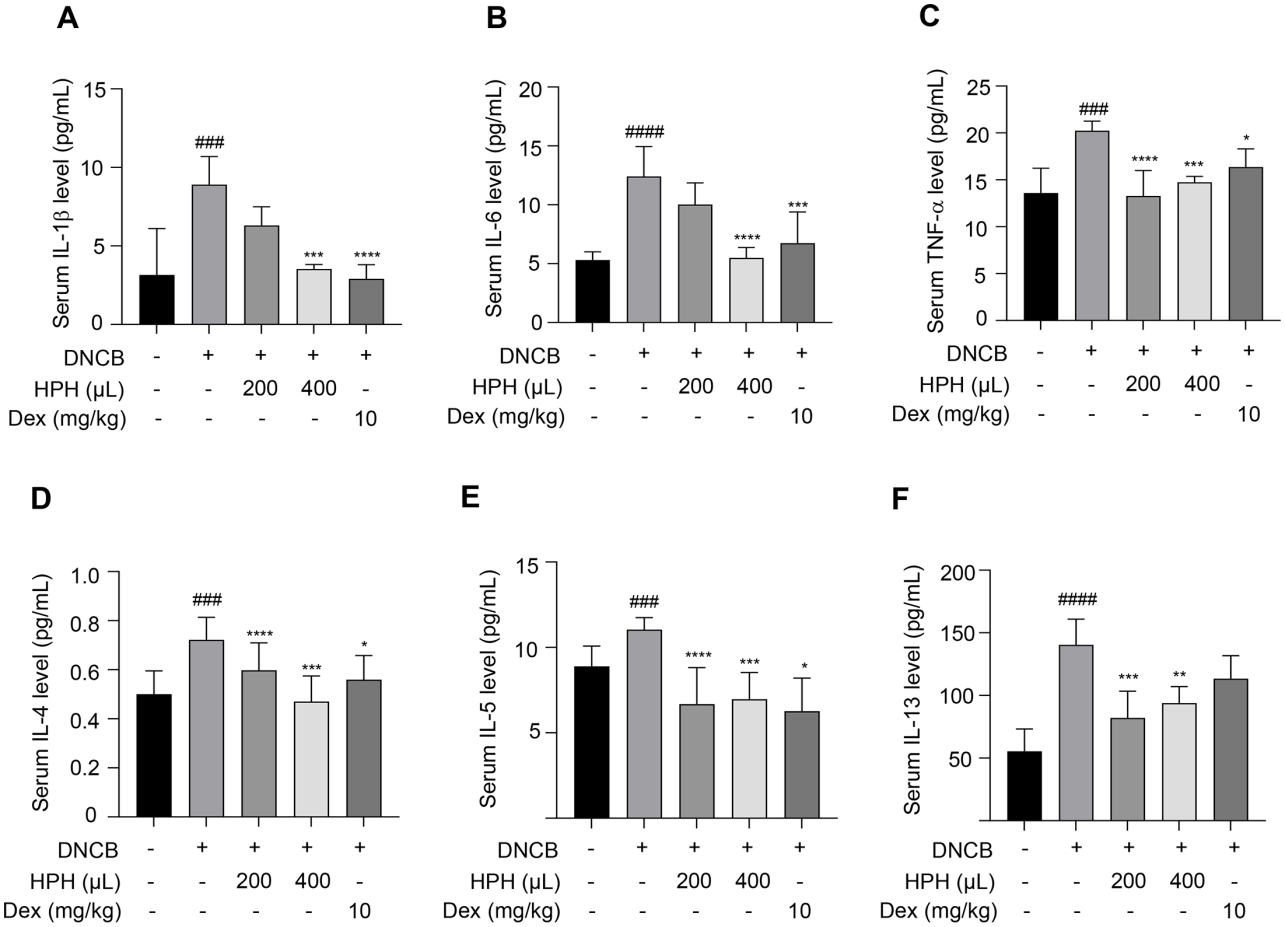
Effect of HPH on pro-inflammatory and Th2 cytokine levels in DNCB-sensitized BALB/c mice. After autopsy, blood samples were collected from individual mice from each group. (**A–C**) Serum pro-inflammatory cytokine (IL- 1β, IL-6, and TNF-α) levels were analyzed using ELISA. (**D–F**) Serum Th2 cytokine (IL-4, IL-5, and IL-13) levels were quantified using ELISA. Data are expressed as the mean ± S.D. *p < 0.05, **p < 0.01, ***p < 0.001, ****p < 0.0001 compared with the control group. ^###^*p* < 0.001, ^####^*p* < 0.0001 compared with the normal group. N, normal group; C, DNCB-induced control group; HPH200, HPH 200 μl-administered group; HPH400, HPH 400 μl-administered group; DEX, dexamethasone 10 mg/kgadministered group.

To investigate the anti-AD effect of HPH, serological analyses were conducted to measure levels of pro-inflammatory and Th2-related cytokines. The levels of Serum IL-1β, IL-6, TNF-α, IL-4, IL-13, and IL-5 levels were quantified. Serum pro-inflammatory and Th2-related cytokine levels, which had been elevated by AD, significantly decreased upon HPH administration (Fig. 6A–6F). Therefore, these findings suggest that HPH plays a crucial role in the systemic regulation of the immune response.

### Changes in the Histological Pathology of DNCB-Sensitized Mice

**Fig. 7:**
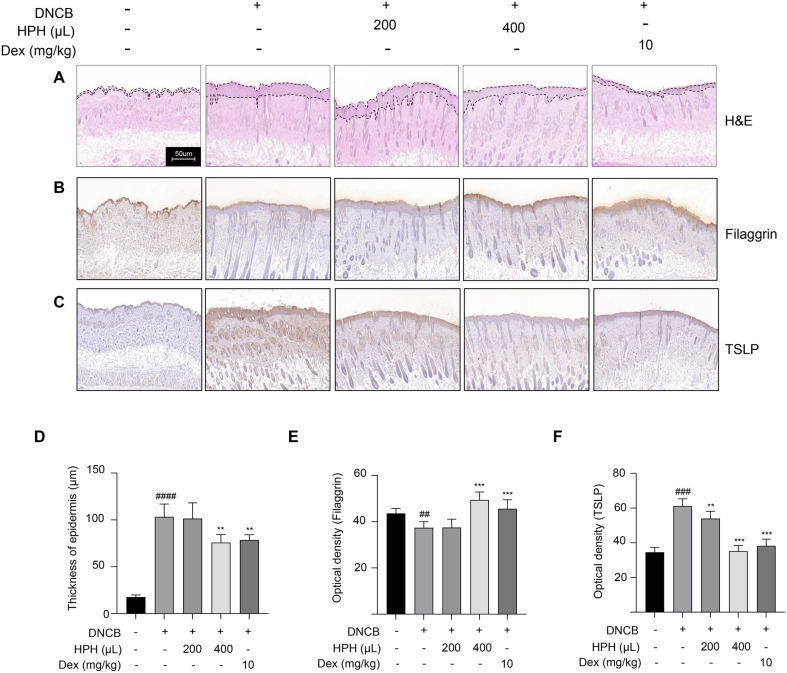
Effect of HPH on dorsal skin barrier in DNCB-sensitized BALB/c mice. After autopsy, dorsal skin tissue samples were obtained from individual mice from each group. (**A**) The typical histological features of the dorsal skin tissues of BALB/c mice in which AD had been induced were visualized using H&E staining. (**B** and **C**) The dorsal skin tissue sections were stained with anti- filaggrin and TSLP antibodies on BALB/c mice in which AD had been induced. (**D**) The epidermal thickness of individual mice from each group was quantified. (**E** and **F**) The filaggrin and TSLP graphs display mean values. In a microscopic image of the dorsal skin tissue, three arbitrary points (left, center, and right) were selected from the entire length of the skin tissue, and images of the points were taken at ×200 magnification. In each of the latter images, two points were arbitrarily selected. In a total of six points/mouse, thickness was measured from the stratum corneum to the basement membrane using i-Solution Lite. Data are expressed as the mean ± SD. **p < 0.01, ***p < 0.001 compared with the control group. ^##^*p* < 0.01, ^###^*p* < 0.001, ^####^*p* < 0.0001 compared with the normal group. N, normal group; C, DNCB-induced control group; HPH200, HPH 200 μl-administered group; HPH400, HPH 400 μl-administered group; DEX, dexamethasone 10 mg/kg-administered group. Scale bar = 50 μm. e, epidermis; d, dermis. Black arrows indicate mast cells.

To evaluate the effect on the skin barrier, tissue sections from each group were stained with H&E, FLG, and TSLP. The DNCB induced AD group exhibited approximately a five-fold increase in epidermal thickness compared to the naïve group; however, this thickening was significantly reduced in the HPH400 group (Fig. 7A and 7D) to a level similar to that of the DEX group. In addition, the expression of skin barrier-related FLG proteins was significantly lower in the DNCB-induced control group compared to the normal group. HPH treatment significantly increased FLG expression relative to the control treatment (Fig. 7B and 7E). Conversely, TSLP expression was increased in the DNCB-induced control group compared to the normal group. HPH treatment decreased TSLP expression in a dose-dependent manner (Fig. 7C and 7F). Overall, these results demonstrate that HPH effectively prevents the deterioration of skin lesions and promotes the recovery of skin to normal conditions.

## Discussion

This study aimed to investigate the efficacy of HPH as a potential treatment for AD using both in vitro and in vivo models. Employing a DNCB-induced mouse model of AD-like skin lesions, our finding indicated that HPH treatment improved skin lesions, reduced the spleen index value, and suppressed the expression of hypersensitivity-related cytokines and chemokines.

These findings were consistent with the results obtained from TNF-α/IFN-γ-stimulated HaCaT cells and splenocytes. Based on the findings, we propose that HPH could serve as a valuable therapeutic agent for hypersensitivity disorders, such as AD.

A recent clinical research has shown that combination therapy incorporating standard AD treatment (*e.g.*, topical glucocorticosteroids, moisturizers, antihistamines, etc.) and HPH was effective in patients with severe AD [34]. As shown in Figs. 4 and 5, HPH exerted a synergistic effect in improving the “Scoring Atopic Dermatitis (SCORAD)” index and reducing serum IgE levels. However, definitive evidence confirming the individual effect of HPH and its underlying mechanisms in the treatment of AD remains limited. Therefore, this study was designed to investigate the mono-therapeutic effects of HPH on AD. We identified mechanisms by which HPH improves the Th1/Th2 imbalance, reduces mast cell numbers, and mitigates IgE levels in response to decreased chemokine and Th2 cytokine levels. In addition, the administration frequency was optimized to be twice weekly to enhance patient compliance and convenience.

DEX is a well-established treatment for AD, yet its long term use has been associated with adverse side effects such as weight loss and muscle atrophy [21, 22]. Previous research by Dong-ho Bak *et al*. demonstrated that HPH can prevent muscle atrophy caused by botulinum toxin muscular injection by inhibiting myostatin expression and ROS generation, suggesting a protective effect against muscle loss [35]. To verify the effect of HPH on muscle loss in the DNCB-induced mouse model of AD-like skin lesions, the muscle mass of mice was measured in this model. Consistent with previous findings, muscle mass decreased by approximately 10% in the DEX and positive control groups compared to the DNCB-induced AD and negative control groups. In contrast, the HPH group showed no significant change in muscle mass (Fig. 4G), indicating that HPH does not cause muscle loss, even with prolonged use in AD treatment.

Mast cells, activated by the cross-linking of allergen-specific IgE antibodies, play a pivotal role in inducing allergic reactions [36]. The interaction between mast cells and IgE mediates scratching behavior, causing dermal damage and subsequent epidermal thickening through enhanced cell division[37]. Therefore, these two symptoms are closely associated, and HPH significantly alleviated both these symptoms.

Th2 cells secrete cytokines, such as IL-4, IL-5, and IL-13 [5-7]. Among these, IL-4 and IL-13 are involved in IgE production in B cells, while IL-5 is essential for eosinophil growth, differentiation, and migration [38]. TARC is a representative Th2 chemokine that promotes the migration and infiltration of Th2 cells, and its serum levels increase in patients with AD [39]. RANTES activates eosinophils and T cells and promotes the adhesion of lymphocytes to the endothelium, causing inflammation [40]. As shown in Fig. 6A–6F, HPH reduced the serum levels of Th2-mediated cytokines (IL-4, IL-5, and IL-13), and chemokines (TARC and RANTES) in the DNCB-induced AD mouse model. This finding suggests that HPH effectively treats AD by inhibiting the migration and infiltration of Th2 cells into the inflammatory sites and restoring the Th1/Th2 balance.

Macrophages are crucial during the inflammatory phase of the immune response, releasing inflammatory cytokines. The F4/80 antigen, a macrophage surface glycoprotein, was reduced in the HPH 200 and HPH 400 groups compared to the control group (Fig. 5B and 5E), indicating that HPH inhibits macrophage infiltration into AD skin lesions [39].

The skin barrier is essential in preventing allergen and microbial entry into the human body [41]. The epidermis acts as both a physical and functional barrier for the human body [42], and defects in this barrier are considered an initial step in the development of AD [43]. FLG is a key epidermal barrier protein that is degraded into free amino acids, which are essential for maintaining skin pH and retaining water, thus contributing to osmolality in the cornified layer [44, 45]. Overexpression of IL-4, IL-13, and TSLP decreases skin FLG levels [46]. In the skin lesion of the HPH400 group, FLG expression increased while TSLP expression decreased, indicating that HPH has a protective effect against AD-induced skin barrier defects. TSLP, which is constitutively secreted by intestinal epithelial cells, can be stimulated by the NF-kB pathway in several epithelial cell lines [47]. In TNF-α/IFN-γ-stimulated HaCaT cells, HPH reversed the TNF-α/IFN-γ-induced phosphorylation of NF-Kb and restored the expression of FLG and TSLP. Collectively, these findings demonstrate that HPH is a promising useful treatment for AD.

## Supplemental Materials

Supplementary data for this paper are available on-line only at http://jmb.or.kr.
