# Supplementary material for: Human Placenta Extract (HPH) Suppresses Inflammatory Responses in TNF-α/IFN-γ-Stimulated HaCaT Cells and a DNCB Atopic Dermatitis (AD)-Like Mouse Model
Source: J Microbiol Biotechnol. 2024 Sep 11;34(10):1969–80. doi: 10.4014/jmb.2406.06045 (PMC11540608; doi:10.4014/jmb.2406.06045)
Supplement: Supplementary file 1 [file jmb-34-10-1969-supple.pdf]

J. Microbiol. Biotechnol. 2024. 34(10): 1969–1980
https://doi.org/10.4014/jmb.2406.06045

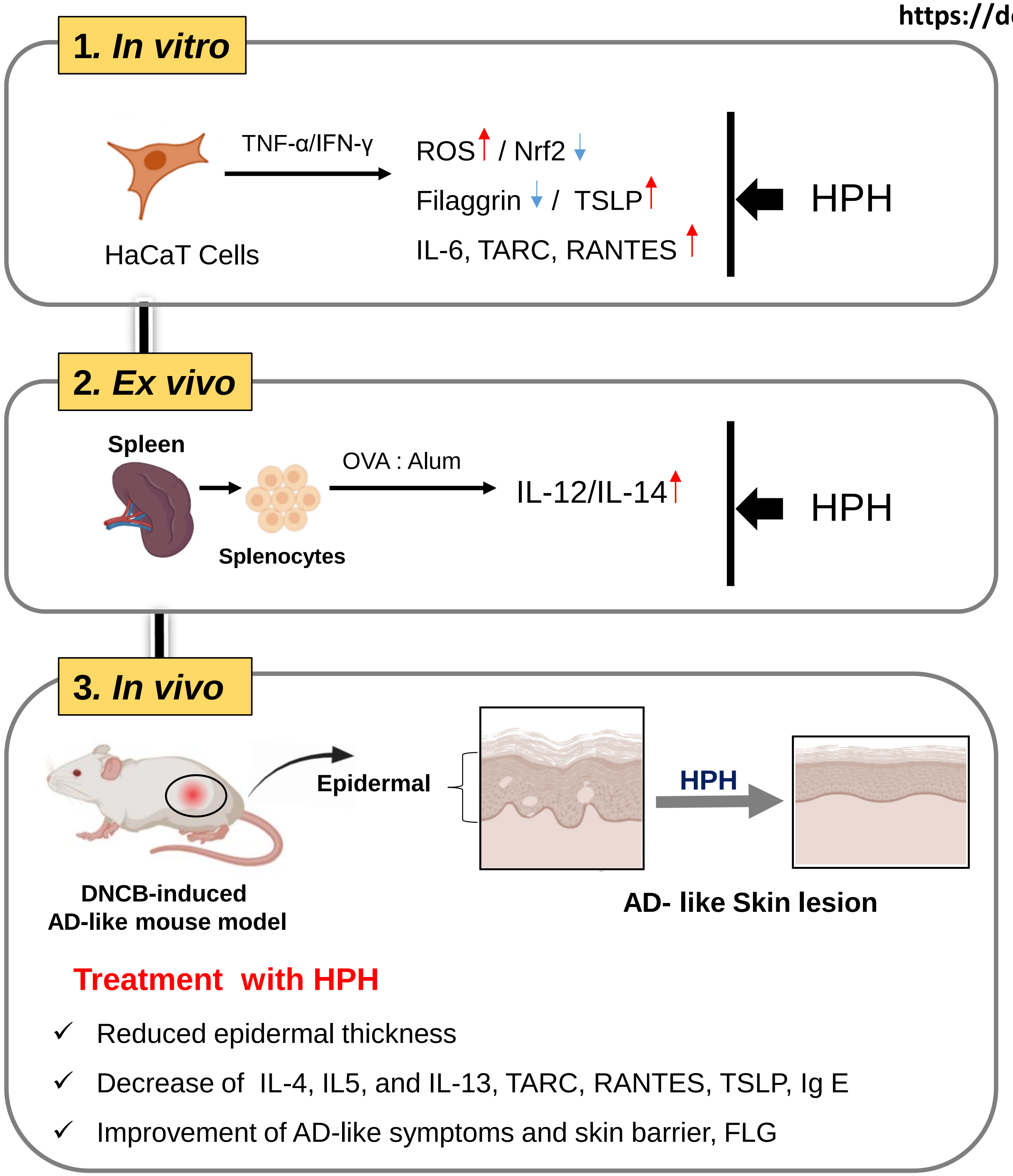

HPH effectively inhibits the development of AD and might be a potentially useful therapeutic agent for AD-like skin disease.
